# The putative tumour suppressor miR-1-3p modulates prostate cancer cell aggressiveness by repressing E2F5 and PFTK1

**DOI:** 10.1186/s13046-018-0895-z

**Published:** 2018-09-05

**Authors:** Sen-Mao Li, Huan-Lei Wu, Xiao Yu, Kun Tang, Shao-Gang Wang, Zhang-Qun Ye, Jia Hu

**Affiliations:** 1 Department of Urology, Institute of Urology, Tongji Hospital, Tongji Medical College, Huazhong University of Science and Technology, Liberalization Ave, No. 1095, Wuhan, 430030 People’s Republic of China; 2 Department of Geriatrics, Tongji Hospital, Tongji Medical College, Huazhong University of Science and Technology, Wuhan, 430030 China; 3 Department of Urology, Peking University First Hospital, Peking University, Beijing, 100034 China

**Keywords:** microRNA, Prostate cancer, Proliferation, Target gene

## Abstract

**Background:**

Previous studies report that miR-1-3p, a member of the microRNA-1 family (miR-1), and functions as a tumor suppressor in several different cancers. However, little is known regarding the biological role and intrinsic regulatory mechanisms of miR-1-3p in prostate cancer (PCa).

**Methods:**

In this study, the expression levels of miR-1-3p were first examined in PCa cell lines and tumor tissues by RT-qPCR and bioinformatics. The in vitro and in vivo functional effect of miR-1-3p was examined further. A luciferase reporter assay was conducted to confirm target associations.

**Results:**

We found that miR-1-3p was significantly downregulated in advanced PCa tissues and cell lines. Low miR-1-3p levels were strongly associated with aggressive clinicopathological features and poor prognosis in PCa patients. Ectopic expression of miR-1-3p in 22RV1 and LncaP cells was sufficient to prevent tumor cell growth and cell cycle progression in vitro and in vivo. Further mechanistic studies revealed that miR-1-3p could directly target the mRNA 3′- untranslated region (3′- UTR) of two central cell cycle genes, E2F5 and PFTK1, and could suppress their mRNA and protein expression. In addition, knockdown of E2F5 and PFTK1 mimicked the tumor-suppressive effects of miR-1-3p overexpression on PCa progression. Conversely, concomitant knockdown of miR-1-3p and E2F5 and PFTK1 substantially reversed the inhibitory effects of either E2F5 or PFTK1 silencing alone.

**Conclusion:**

These data highlight an important role for miR-1-3p in the regulation of proliferation and cell cycle in the molecular etiology of PCa and indicate the potential for miR-1-3p in applications furthering PCa prognostics and therapeutics.

**Electronic supplementary material:**

The online version of this article (10.1186/s13046-018-0895-z) contains supplementary material, which is available to authorized users.

## Background

MicroRNAs (miRNAs) belong to a class of conserved and small noncoding RNAs that repress protein expression through base pairing with the 3′-untranslated region (3’-UTR) of target mRNA [1, 2]. It is well documented that miRNA aberrations may be an important factor in cancer development. The potential connection between miRNA regulation and cancer has been made at several levels, suggesting that miRNAs play critical roles in cellular growth and differentiation, which are two cellular processes that are commonly defective in tumor cells [3–6].

Prostate cancer (PCa) is one of the most frequently diagnosed cancers among men and is the third leading cause of male cancer-related death in the United States [7]. Despite the initial success of surgery and radiation therapy for localized prostate cancer, > 30% patients experience biochemical recurrence and emergence of advance-stage disease particularly, metastatic progression [8–10]. Therefore, a more thorough understanding of the mechanisms underlying PCa pathogenesis will help to develop more effective therapeutic strategies, for which there is an urgent need. Alteration of miRNA expression is observed in PCa that have been collected from different study cohorts [11–13]. Furthermore, several miRNAs (eg, miR-1, miR-135a, miR-21, miR-96, miR34a, miR-203 and miR-205) have been shown to regulate PCa cell growth, apoptosis, migration and/or invasion [9, 14–17], suggesting a dysfunction of miRNA may be associated with prostate carcinogenesis. Clearly, more comprehensive research is required to elucidate the role of miRNAs during PCa progression and to identify those miRNAs that could serve as novel prognostic predictors and therapeutic targets for PCa.

Previous profile studies of miRNA expression have noted the downregulation of a series of miRNAs,including miR-1-3p in PCa tissues. It has been shown that ectopic expression of miR-1 inhibits prostate cancer cell growth, epithelial-mesenchymal transition and bone metastasis [18, 19]. In addition, miR-1-3p has been reported to suppress tumor growth in colon carcinomas [20], decrease cellular proliferation and migration of oral squamous cell carcinoma [21], inhibit cell proliferation and invasion and induce apoptosis in bladder cancers [22]. These data indicate a potential tumor suppressive function of miR-1-3p. However, the role of miR-1-3p in prostate carcinogenesis and the molecular mechanisms by which it functions and modulates the malignant phenotypes of PCa cells remain to be delineated.

In this study, we report that deregulation of miR-1-3p in PCa is important in the development of an aggressive phenotype and is correlated with a poor prognosis. Ectopic overexpression of miR-1-3p in PCa cells is sufficient to inhibit cell invasion, both in vitro and in vivo. More importantly, for the first time, we provide evidence that miR-1-3p directly targets two central cell cycle genes, the E2F transcription factor 5 (E2F5) and PFTAIRE Protein Kinase 1 (PFTK1) mRNA, to suppress cell proliferation. Collectively, the results of this study provide an explanation for the aggressiveness of PCa and link it mechanistically to interactions between miR-1-3p, E2F5 and PFTK1. Our results also suggest that miR-1-3p could be employed as a new prognostic marker and/or as an effective therapeutic target for PCa.

## Methods

### Patients and tissue samples

PCa samples and adjacent normal tissue samples were collected during radical prostatectomy from PCa patients between 2008 and 2014 at the Tongji Hospital, Tongji Medical College, Huazhong University of Science and Technology in Wuhan, China. The PCa cases selected were based on a clear pathological diagnosis, follow-up data, and absence of androgen deprivation therapy, chemotherapy, radiotherapy or other anticancer treatment before surgery. All specimens had confirmed pathological diagnosis and were classified according to the WHO criteria. The clinicopathological patient information was collected and summarized in Table 1. All protocols were approved by the Ethics Committee of Tongji Hospital, Tongji Medical College, Huazhong University of Science and Technology, and informed consent was obtained from all patients before surgery. All in vivo protocols were approved by the Institutional Animal Care and Use Committee of Tongji Hospital, Tongji Medical College, Huazhong University of Science and Technology.

**Table 1:** Relationship between miR-1-3p and clinicopathologic variables in patients with prostate cancer

Variables	miR-1-3p			*P* value
	All cases	Low expression	High expression	
	(*n* = 124)	(*n* = 54)	(*n* = 70)	
Age				
≥ 65y	70	30 (42.9%)	40 (57.1%)	0.860
< 65y	54	24 (44.4%)	30 (55.6%)	
Serum PSA				
≥ 10 ng/ml	74	35 (47.3%)	39 (52.7%)	0.306
< 10 ng/ml	50	19 (38.0%)	31 (62.0%)	
Gleason score				
≥ 7	76	42 (55.3%)	34 (44.7%)	0.001
< 7	48	12 (25.0%)	36 (76.0%)	
pT stage				
< T3	35	23 (65.7%)	12 (34.3%)	0.002
≥ T3	89	31 (34.8%)	58 (65.2%)	
Lymph node metastasis				
Presence	31	20 (64.5%)	11 (35.5%)	0.012
Absence	93	34 (36.6%)	59 (63.4%)	
Seminal vesicle invasion				
Presence	33	19 (57.6%)	14 (42.4%)	0.164
Absence	81	35 (43.2%)	46 (56.8%)	
Biochemical recurrence				
Presence	14	9 (64.3%)	5 (35.7%)	0.097
Absence	110	45 (40.9%)	65 (59.1%)	

### Bioinformatics analysis databases

The RNA-seq data were from TCGA and downloaded from the TCGA Data Portal (https://portal.gdc.cancer.gov/). The miRNA target predicting algorithm TargetScan Release 7.1 (http://www.targetscan.org/vert_72) was used to predict miRNAs targeting E2F5 and PFTK1 and their binding regions.

### Cell culture

22RV1 and LNcaP cells (ATCC) were maintained in RPMI-1640 medium (HyClone, Logan, UT, USA) supplemented with 10% fetal bovine serum (FBS) (Gibco) and the normal prostate epithelial cells RWPE-1 (ATCC) were maintained in Keratinocyte-SFM (Gibco, GrandIsland, NY, USA). All cells were cultured in a humidified atmosphere of 5% CO2 maintained at 37 °C.

### Oligonucleotide, lentivirus production and cell transfection

All small RNA molecules were ordered from RiboBio Co., Ltd.(Guangzhou, China), including miR-1-3p mimics, mimics negative controls (mimics-NC), miR-1-3p inhibitor, inhibitor negative controls (inhibitor-NC),siE2F5 (sense:5′-CAGAUGACUACAACUUUAATT-3′; antisense:5′-UUAAAGUUGUAGUCAUCUGTT-3′) and siPFTK1 (sense: 5’-GTTCATTCTTTACCACATT-3′;antisense: 5’-AGGTTGCATCTTTGTTGAA-3′). MiR-1-3p mimics are double-stranded RNA molecules containing the miR-1-3p sequence, while miR-1-3p inhibitors are single stranded RNA molecules containing the miR-1-3p reverse complement sequence, which can competitively bind to endogenous miR-1-3p. For lentiviral-mediated overexpression, viral particles were harvested 48 h after transfection of 293FT cells with pCDH-CMV-miR-1-3p, –E2F5 or –PFTK1 and the packaging plasmids pRSV/pREV, pCMV/pVSVG and pMDLG/pRRE using Lipofectamine 2000 (Invitrogen). MiR-NC (TTCTCCGAACGTGTCACGT) was cloned into the same backbone and the resulting construct Lenti-miR-NC served as a negative control. The transfection or infection efficiencies were detected by RT-qPCR. Recombinant lentivirus-transducing units were used to infect LNcaP cells in the presence of 8 mg/ml Polybrene (Sigma, St Louis, MO, USA). Cells were plated in growth medium at a density of 45% to 70%. The transfection was carried out using Lipofectamine RNAiMax (Invitrogen, Carlsbad, CA, USA) 24 h later according to the manufacturer’s protocol. The final concentration of RNAs was 75 nM for each well. All cell lines were tested and found to be free of mycoplasma contamination.

### RNA isolation and quantitative real-time PCR

Total RNA of cells was extracted with TRIzol reagent (Invitrogen, Carlsbad, CA) according to the manufacturer’s protocol. Reverse transcription of microRNA and mRNA were done using RevertAid™ First Strand cDNA Synthesis Kit (Fermentas, Vilnius, Lithuania) and miProfile™ miRNA qPCR Primer (GeneCopoeia, Guangzhou,China). RT-qPCR analysis of miRNA was performed with the Platinum SYBR Green qPCR Supermix UDG kit (Invitrogen, Carlsbad, CA) using synthesized primers from GeneCopoeia (Guangzhou, China). The U6 primers were obtained from GeneCopoeia. All experiments were done in triplicate. The expression level values were normalized to those of the small nuclear RNA U6 as a control. Several primer sequences used are available as follows:

GAPDH primers:

- Forward: 5’-TCCCATCACCATCTTCCA-3’
- Reverse: 5’-CATCACGCCACAGTTTCC-3’

E2F5 primers:

- Forward: 5’-CACCTTCTGGTACACAACTGG-3’
- Reverse: 5’-GGGCTTAGATGAACTCGACTC-3’

PFTK1 primers:

- Forward: 5’-TTACATCCACCAGCGTTA-3’
- Reverse: 5’-TTGGAGTATGTGTGGCTA-3’

CDK4 Primers:

- Forward: 5’-ATGGCTACCTCTCGATATGAGC-3’
- Reverse: 5′- CATTGGGGACTCTCACACTCT-3’

CDK2 Primers:

- Forward: 5′- GCGAATTCCCCAGCCCTAATCTCA-3’
- Reverse: 5′- GCCTCGAGAACCCTCTTCAGCAATAA-3′

### Colony formation and MTS cell proliferation assay

The colony formation assay was conducted as previously described. Briefly, exponentially growing cells were plated at approximately 2000 cells per well in 6-well plates after transfection. Culture medium was changed every 3 days. Colony formation was analyzed 12 days following infection by staining cells with 0.05% crystal violet solution for 30 min. The number of colonies was counted using an inverted microscope (Olympus, Japan). Cell proliferation was assessed by using the CellTiter 96 Aqueous One Solution Cell Proliferation. Assay kit (Promega, Madison, WI, USA) as previously described. Briefly, RNA transfected cells were grown in 96-well plates at a density of 2000 cells/well. Cell growth was measured daily for 4 days. At each time point, 20 μl of CellTiter 96 Aqueous One Solution was added and incubated. Absorbance was detected by a microplate reader (Bio-Rad, Berkeley, CA, USA) at 490 nm.

### Cell cycle

At 72 h after transfection, cells were fixed in 70% cold ethanol, incubated with RNase A (Sigma,St. Louis, MO, USA) and stained by propidium iodide (PI) (Nanjing KeyGen Biotech Co., Ltd., Nanjing, China) staining solution. After staining, the cells were analyzed on a FACSort flow cytometer (BD Biosciences, San Diego, CA, USA). The data were processed by CELL quest software (BD Biosciences).

### Western blot analysis

Cells were harvested at 72 h following transfection. Proteins were separated by 10% SDS/PAGE and transferred onto PVDF membranes (Millipore, Billerica, MA, USA). After blocking, the membranes were incubated overnight at 4 °C with appropriate dilutions of specific primary antibodies as follows: E2F5 (1:1000) (Abcam, ab44996), PFTK1 (1:1000) (Abcam, ab104150), CDK4 (1:1000) (Cell Signaling Technology, 12,790), CDK2 (1:1000) (Cell Signaling Technology, 2546), GAPDH (1:500) (Boster, Wuhan, China). Next, membranes were incubated with corresponding second antibody and detected by enhanced chemiluminescence (ECL) assay kit (Millipore).

### Luciferase reporter assay

E2F5 and PFTK1 3’UTR reporter and control constructs were purchased from GENECHEM. Tumor cells overexpressing miR-1-3p and miR-NC cultured in 48-well plates were co-transfected with 1.5 mg of firefly luciferase reporter and 0.35 ng Renilla luciferase reporter with Lipofectamine RNAiMax (Invitrogen, Carlsbad, CA, USA). 24 h post transfection, firefly luciferase activities were measured using the Dual Luciferase Assay (Promega) and the results were normalized with Renilla luciferase according to the manufacturer’s protocol.

### Xenograft model of PCa in nude mice

Two groups of five male BALB/c nude mice at 6 weeks of age each were injected subcutaneously with prepared cells (1 × 10^6^ LNcaP cells stably expressing miR-1-3p or miR-NC) at the same site. Tumor onset was measured with calipers at the site of injection every 3–4 days by two trained laboratory staff members at different times on the same day, starting 12 days after injection when appreciable tumor formed subcutaneously. Tumor volume was calculated using the formula, V = 0.5ab^2^, where a represents the larger and b represents the smaller of the two perpendicular indexes. Animals were euthanized and xenografts were harvested 40 days after injection and tumors were weighed. Formalin-fixed, paraffin-embedded PCa xenografts were assessed by hematoxylin and eosin (HE) and Ki-67 staining and evaluated for target gene expression. Nude mice were manipulated and cared for according to NIH Animal Care and Use Committee guidelines in the Experiment Animal Center of the Tongji Medical College of Huazhong University of Science and Technology, China.

### Immunohistochemistry (IHC)

Formalin-fixed, paraffin-embedded tissue sections (5 μm) were deparaffinized in xylene and rehydrated with gradient concentrations of ethanol. The tissue sections were stained with specific antibodies against E2F5 (1:400) (Abcam, ab203124) and PFTK1 (1:400) (Abcam, ab224098). Sections incubated with secondary antibodies in the absence of primary antibodies were used as negative control. Hematoxylin was used for counterstaining. Slides were viewed and photographed under a light microscope.

### Statistical analysis

Statistical analysis was performed using SPSS software (SPSS Standard version 19.0, SPSS Inc. Chicago, IL). Differences between variables were assessed by the χ2 test or Fisher’s exact test. Continuous data were compared using the Student’s two-tailed t test. For survival analysis, Kaplan-Meier method, and log-rank tests were used to identify the potential differences between progression-free-survival in the two patient cohorts. Hazard ratios (HRs) were estimated with a multivariate Cox proportional hazards model. X-tile software version 3.6.1 (Yale University School of Medicine, New Haven, CT, USA) with a built-in validated feature was used to define the cutoff point [23]. Data are represented as the mean ± SEM. In all cases, *p*-values < 0.05 were considered to be statistically significant.

## Results

### Levels of miR-1-3p are frequently lower in PCa cell lines and tissues, and low miR-1-3p expression is associated with a poor PCa prognosis

**Fig. 1:**
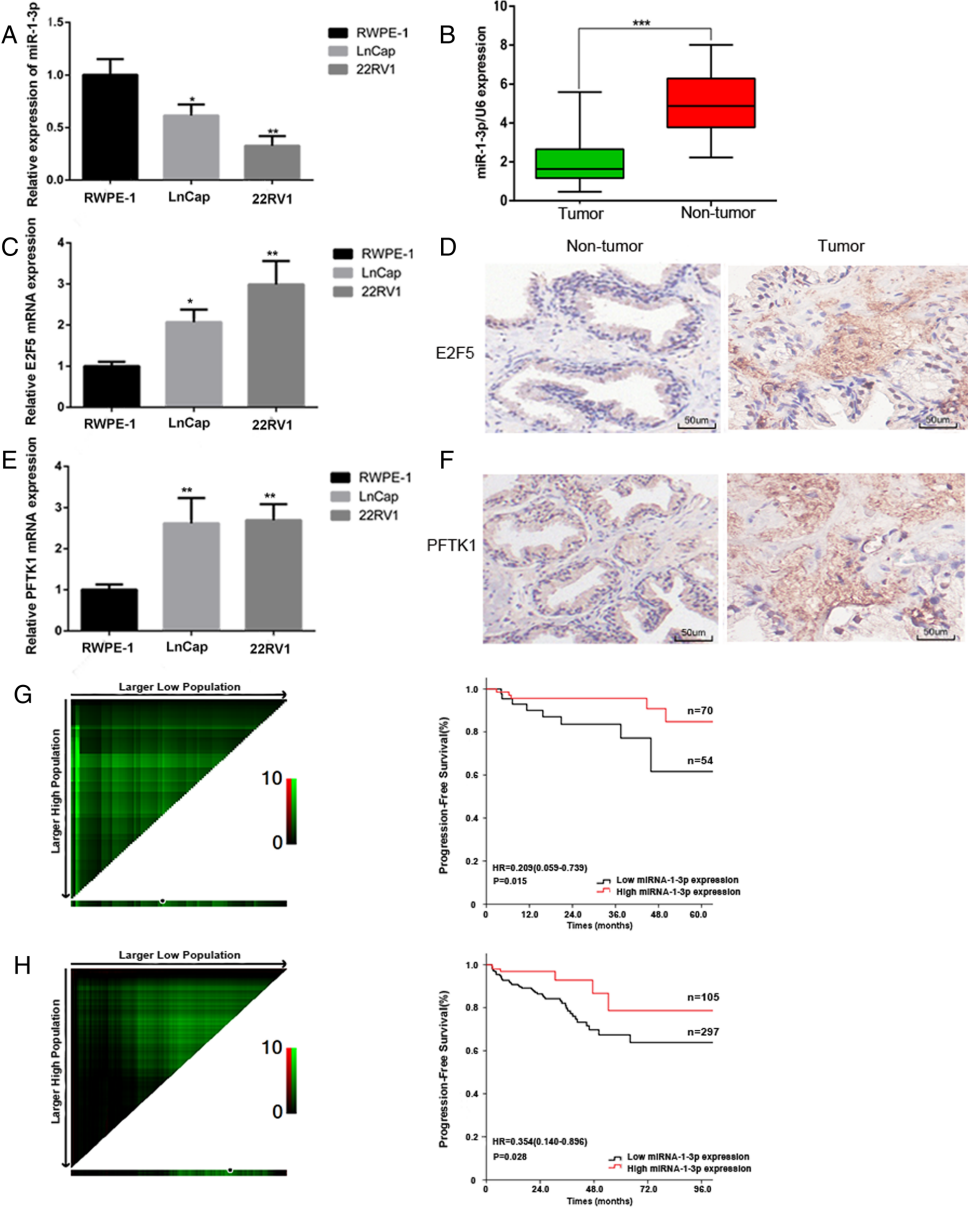
Mature miR-1-3p expression levels in prostate cancer cell lines and tissues, and the prognostic value of miR-1-3p levels in patients with PCa. **a** Expression levels of miR-1-3p, (**c**) E2F5 and (**e**) PFTK-1 were examined by RT-qPCR in RWPE-1 cells and two PCa cell lines. GAPDH and U6 served as corresponding loading controls. Error bars represent the mean ± S.D. of three independent experiments (*P < 0.05, **P < 0.01 and ***P < 0.001 compared to RWPE-1 group). **b** Expression levels of mature miR-1-3p in 25 paired PCa and adjacent non-tumour tissues. Alteration of expression is shown as box plot presentations, with the y axis indicating miR-1-3p expression. The mean level of miR-1-3p expression in PCa tissues were significantly lower than that in non tumor tissues. (***P < 0.001, independent t test). **d** E2F5 and (**f**) PFTK-1 protein expression in primary prostate tissues was detected by Immunohistochemically staining assay. Scale bar: 50 μm. **g** 124 PCa patients from the Tongji Hospital, Tongji Medical College, Huazhong University of Science and Technology, and (**h**) 402 PCa patients from the TCGA database. Left panel: X-tile plots automatically selected the cut-off point of miR-1-3p. Right panel: Kaplan–Meier analysis of survival and the COX proportional hazards model for the hazard ratio

To identify the role of miR-1-3p in tumorigenesis and progression of PCa, we first analyzed miR-1-3p expression by RT-qPCR in three prostatic cell lines and 25 pairs of PCa and corresponding adjacent non-tumor tissues. The results showed that both PCa cell lines (22RV1, LNcaP) had lower levels of miR-1-3p expression than that in the normal prostatic cell line RWPE-1 (Fig. 1a). In primary PCa samples, 17/25 (68%) of cases had miR-1-3p levels reduced by more than half, compared with adjacent normal prostate tissue samples (Fig. 1b). Moreover, we evaluated the E2F5 and PFTK1 mRNA and protein levels by RT-qPCR and IHC, respectively. An inverse correlation between miR-1-3p and E2F5 (Fig. 1c-d) and PFTK1 (Fig. 1e-f) expression levels was discovered in prostate cell lines and tissues. To further investigate the clinicopathological and prognostic significance of miR-1-3p levels in PCa patients, the levels of miR-1-3p in a large cohort of 124 PCa tissues (including the 25 samples used before) were examined by RT-qPCR. The cut-off point for dividing tumors into groups of low or high-expression of miR-1-3p was determined using X-tile software (Yale University School of Medicine, New Haven, CT, USA). In multivariable analysis, the low-level expression of miR-1-3p was associated with a short progression-free survival time both the Tongji cohort (*p* = 0.015, HR = 0.209 (0.059–0.739)) (Fig. 1g) and the TCGA database (*p* = 0.028, HR = 0.354(0.140–0.896)) (Fig. 1h), after adjusted for potentially predictive factors such as age, PSA, pT stage, lymph node metastasis (Additional file 1: Table S1).

### Overexpression of miR-1-3p inhibits the proliferation of PCa in vitro

To further explore the biological role of miR-1-3p in PCa, we performed gain- and loss-of-function experiments by transfecting cells with a miR-1-3p mimic or inhibitor targeting miR-1-3p (miR-1-3p inhibitor). dsControl was used as a nonspecific control.

**Fig. 2:**
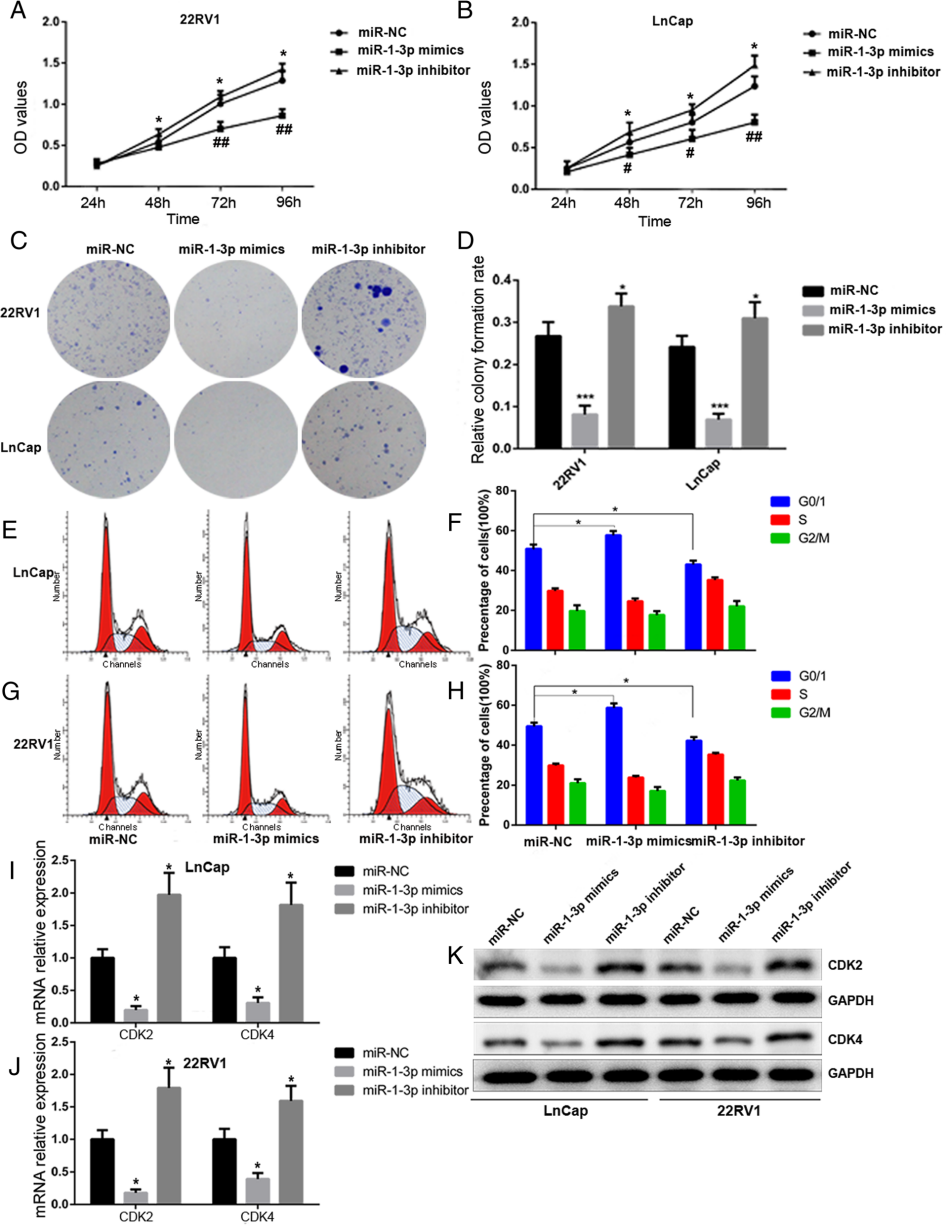
MiR-1-3p attenuates prostate cancer cell lines proliferation through inducing cell cycle arrest at G0/G1 phase. LnCap and 22RV1 cells were transfected with 100 nM indicated RNAs molecules respectively for 72 h. **a** and **b** MTS assays revealed cell growth curves of both PCa cells in every 24 h. **c** Representative micrographs and (**d**) relative quantification of crystal violet-stained cell colonies analyzed by clonogenic formation. **e** and **f** flow cytometric determination of proportion of LnCap and 22RV1 cells in distinct cell cycle phases. **g** and **h** Expression of CDK2 and CDK4 mRNA in LnCap and 22RV1 cells were assessed by RT-qPCR following transfection with 100 nm miR-1-3p mimics, miR-1-3p inhibitor, or their negative controls (NC). GAPDH served as a loading control. **i** Western blot analysis of the relative expression of CDK2 and CDK4 in response to indicated RNAs molecules. GAPDH served as a loading control. The results were plotted as the mean ± S.D. of three independent experiments. (*P < 0.05, **P < 0.01, ***P < 0.001, ^#^ *P* < 0.05 and ^##^ *P* < 0.01)

The efficiency of miR-1-3p upregulation by the miR-1-3p mimic, and knockdown by the miR-1-3p inhibitor was confirmed by quantifying the transcription levels of miR-1-3p with RT-qPCR (Additional file 2: Figure S1). As shown in Fig. 2a-b, reduced expression of miR-1-3p enhanced proliferation, while induced expression of miR-1-3p inhibited proliferation of 22RV1 and LNcaP cells compared with the control cells transfected with dsControl. The PCa suppressing role of miR-1-3p was confirmed by the results of the clonogenic assay showing that knockdown of miR-1-3p increased colony numbers in both PCa cell lines (Fig. 2c-d). To investigate the mechanism by which overexpression of miR-1-3p blocks PCa cell proliferation, we examined whether growth inhibition was associated with cell cycle dysfunction. The effect of miR-1-3p on the cell cycle distribution in 22RV1 and LNcaP cells were examined by flow cytometric analysis. Compared with miR-NC transfected cells, miR-1-3p transfected cells showed a marked increase in the number of cells in the G0/G1 phases (Fig. 2e-f). Next, we measured the molecular expression of cell-cycle related proteins, cyclin dependent kinase 2 (CDK2) and cyclin dependent kinase (CDK4), which regulate progression through the cell cycle. The activity of these kinases are especially critical during the G1 to S phase transition. As illustrated in Fig. 2g-i, our data showed that the gene and protein expression for CDK2 and CDK4 are significantly decreased in the miR-1-3p-activated cells, and increased in miR-1-3p-silenced cells compared with control cells. Collectively, these results demonstrate that ectopic expression of miR-1-3p inhibits PCa cell proliferation and induces G0/G1 cell cycle arrest.

### MiR-1-3p directly targets E2F5 and PFTK1

**Fig. 3:**
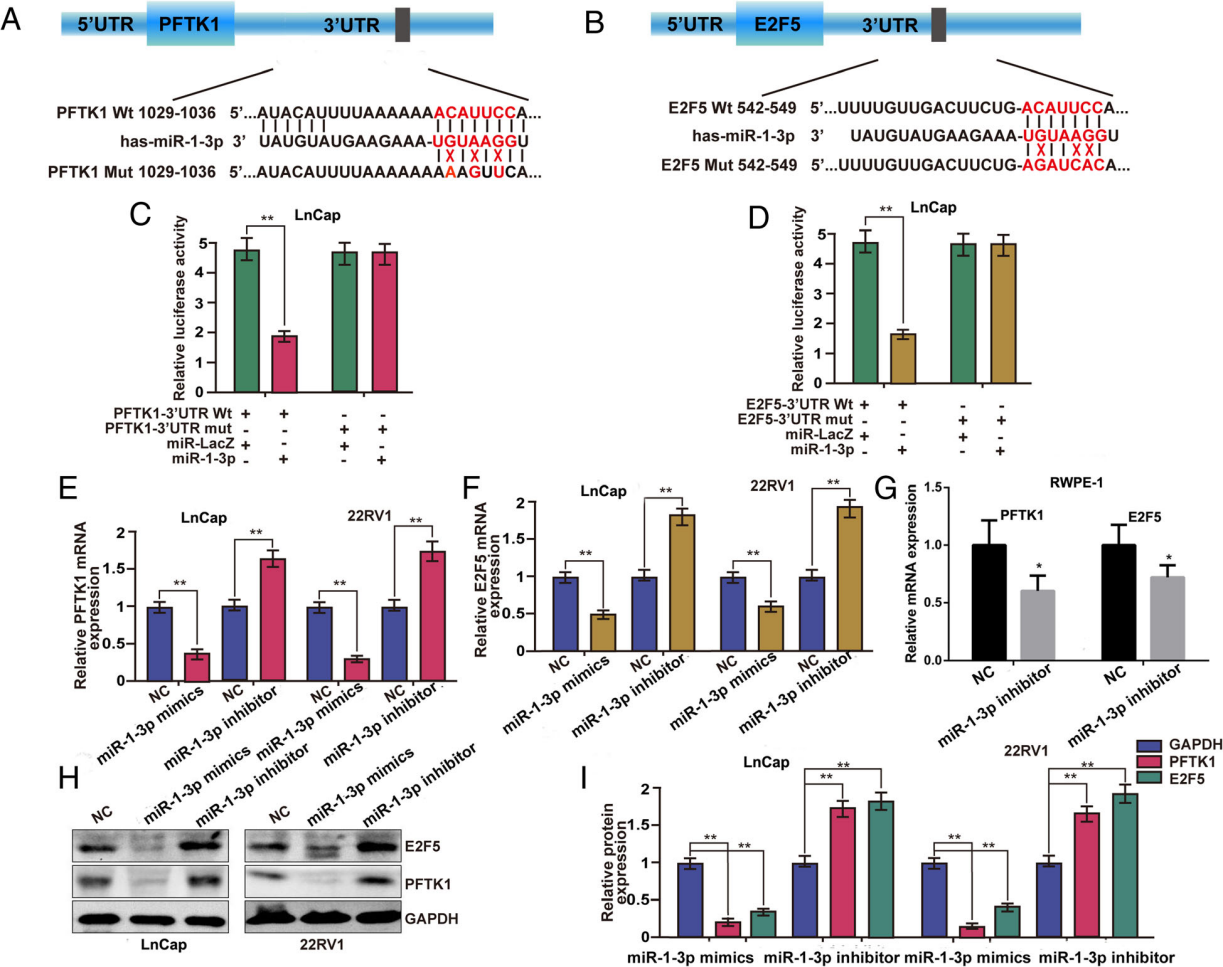
E2F5 and PFTK-1 were identified as direct target genes of miR-1-3p and down-regulated by miR-1-3p in the prostate cancer cell. **a** and **b** Schematic diagram of the predicted target binding sites of miR-1-3p in the 3′-UTR of E2F5 and PFTK-1. The seed recognition site is denoted. All nucleotides of the 3′-UTR region of E2F5 and PFTK-1 that binds with miR-1-3p are highly conserved across species as predicated by TargetScan (http://www.targetscan.org/vert_72/). **c** and **d** The luciferase activity of the wild type E2F5/ PFTK-1 3’-UTR (Wt) and mutant E2F5/ PFTK-1 3’-UTR (Mut) co-transfected with miR-1-3p mimics or a miRNA negative control (miR-LacZ) was measured in LnCap cells. Relative luciferase activity was plotted as the mean ± S.D. of three independent experiments. **e**-**g** Expression of PFTK-1 and E2F5 mRNA in LnCap, 22RV1 and RWPE-1 cells were assessed by RT-qPCR following transfection with miR-1-3p mimics, miR-1-3p inhibitor, or their negative controls (NC). GAPDH served as a loading control. **h** and **i** Western blot analysis of the protein levels and relative expression of E2F5 and PFTK-1 in response to 100 nM of indicated RNAs molecules. GAPDH served as a loading control in LnCap and 22RV1 cells. (*P < 0.05, **P < 0.01)

To elucidate how miR-1-3p blocks G0/G1 phase progression and cell growth, we used TargetScan7.1 algorithms, a bioinformatic tool for microRNA target prediction. We identified E2F5 and PFTK1, two central cell cycle genes among the list of predicted targets of miR-1-3p (Fig. 3a-b). E2F5 belongs to E2F family and has been reported to be involved in the promotion of cell proliferation and cell cycle progression [24]. PFTK1 is a new member of the CDK family. Recent reports demonstrated that PFTK1 participates in cell cycle regulation by accelerating the G0/G1–S phase transition [25]. We further experimentally validated whether E2F5 and PFTK1 were the direct targets of miR-1-3p by employing a dual-luciferase reporter system. We subcloned the 3’-UTRs of the E2F5 mRNA and PFTK1 mRNA, including the predicted miR-1-3p recognition site (Wt) or the mutated sequences (Mut) in the pGL3 vector downstream of the luciferase open reading frame. MiR-LacZ was used as a miRNA blank vector control. As predicted, miR-1-3p inhibited the activity of luciferase of the wild-type but not mutant 3’-UTR of E2F5 and PFTK1 in LNcaP cells (Fig. 3c-d). This further confirms that miR-1-3p down-regulates expression of E2F5 and PFTK1 in PCa. Both gain-of-function and loss-of-function analyses support the suppressive effect of miR-1-3p on the expression of E2F5 and PFTK1 mRNA in 22RV1 and LNcaP cells (Fig. 3e-f). E2F5 and PFTK1 mRNA expression levels in the normal prostate epithelial cell line RWPE-1 were also tested after transfecting the miR-1-3p inhibitor, and the results were the same as in the PCa cell lines (Fig. 3g). In addition, a similar suppressive effect on the E2F5 and PFTK1 protein level was also observed in both PCa cells (Fig. 3h-i). Therefore, these results indicate that miR-1-3p inhibits cell proliferation by targeting and suppressing E2F5 and PFTK1 in PCa.

### E2F5 and PFTK1 function to promote PCa cell proliferation and cell cycle progression

**Fig. 4:**
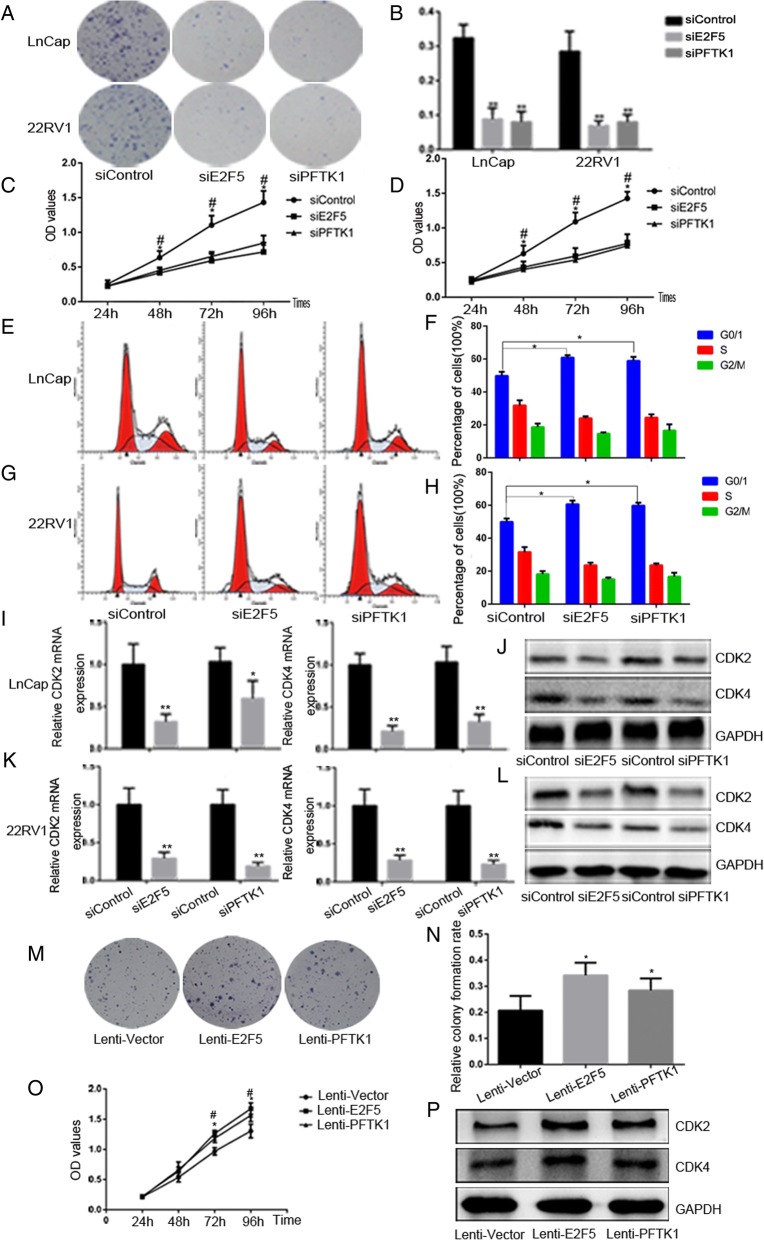
E2F5 and PFTK1 are involved to promote PCa cell proliferation and cell cycle progression in vitro*.* LnCap and 22RV1 cells were transfected with siRNAs of E2F5 and PFTK-1 respectively for 72 h; siControl served as negative control. **a** Representative photographs of colony formation assay and (**b**) quantification of the cell colonies formation were used to determine the proliferative ability of PCa Cells. **c** and **d** MTS assays revealed cell growth curves of indicated cells in every 24 h. **e** and **f** Flow cytometric determination of proportion of indicated cells in distinct cell cycle phases. **g-j** RT-qPCR and Western blot analysis the gene and protein expression of CDK2 and CDK4. LnCap cells were infected by lenti-E2F5 and PFTK1 to overexpress E2F5/PFTK1. Lenti-vector served as negative control. **k** and **l** The cell colony formation was used to determine the proliferative ability. **m** MTS assays revealed cell growth curves in every 24 h. **n** Western blot analysis the expression of CDK2 and CDK4 indicated cells. The results were plotted as the mean ± SEM of three independent experiments, with at least three replicates in each independent experiment. (*P < 0.05, **P < 0.01; #*P* < 0.05, ##*P* < 0.01)

To determine the biological functions for E2F5 and PFTK1, we performed loss-of-function experiments by knocking down E2F5 and PFTK1 using siRNA in 22RV1 and LNcaP cells. The knockdown efficiencies were confirmed by measuring protein levels by WB (Additional file 3: Figure S2). The results of the colony formation assays showed that the proliferation capacities of 22RV1 and LNcaP cells were significantly reduced after treatment with siRNAE2F5 and siRNAPFTK1 (Fig. 4a-b). MTS assays further confirmed significant inhibition of cell growth in both PCa cell lines following E2F5 and PFTK1 silencing (Fig. 4c-d). Consistently, FACS and WB results indicated that the proportion of cells in the G0/G1 phases was significantly increased (Fig. 4e-f) and the expression of CDK2 and CDK4 was inhibited (Fig. 4g-j) in E2F5 and PFTK1-silenced PCa cells. Next, we performed gain-of-function experiments to further elucidate the effects of PFTK1 and E2F5. As expected, stable overexpression of PFTK1/E2F5 can induce more colony formation (Fig. 4k-l), increase cell proliferation (Fig. 4m) and led to upregulation of CDK2 and CDK4 in LNcaP cells (Fig. 4n). Altogether, these data demonstrate that E2F5 and PFTK1 promote PCa cell proliferation, and silencing them as effectively as miR-1-3p overexpression.

**Fig. 5:**
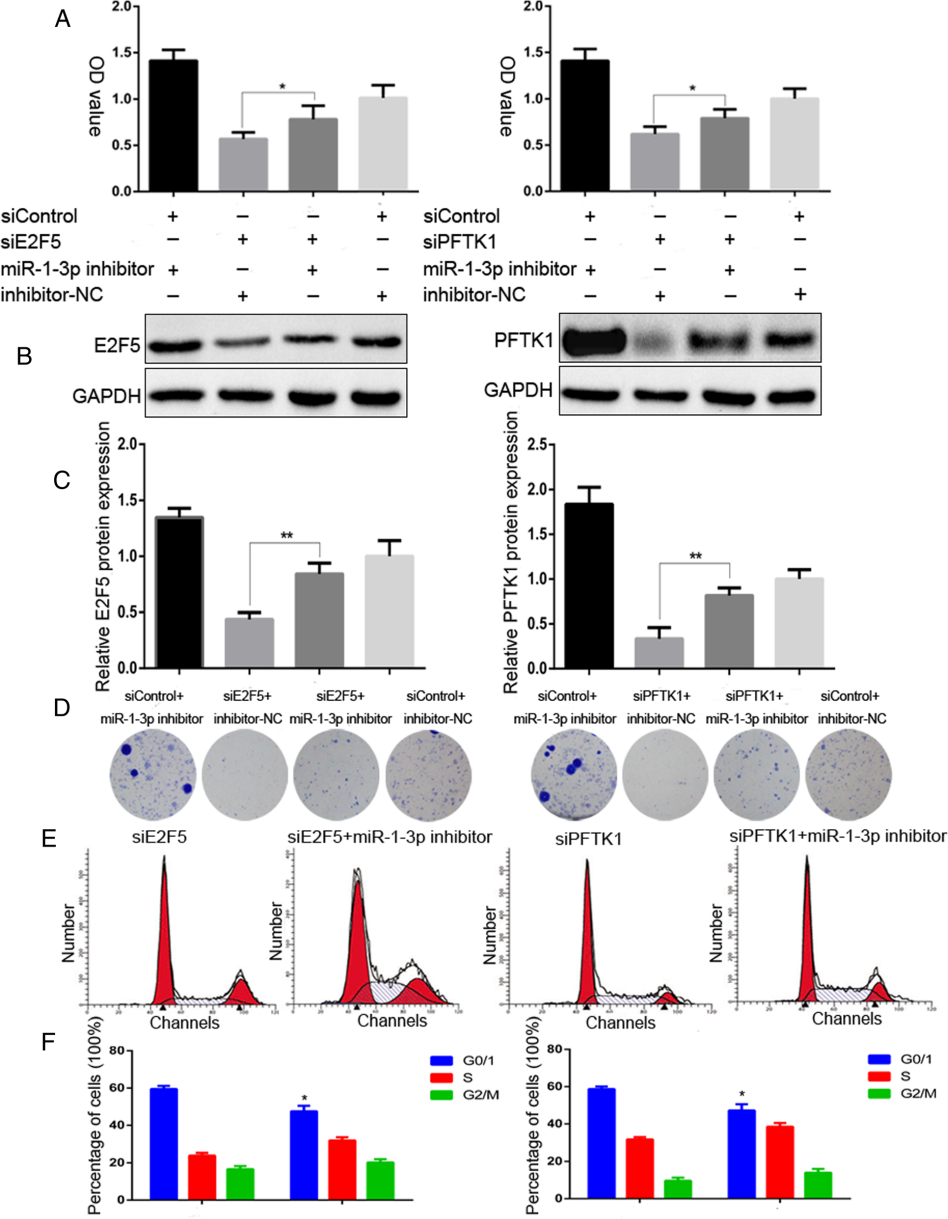
E2F5 and PFTK1 are a functional targets of miR-1-3p suppression of PCa cells proliferation and cell cycle progression. LnCap cells were co-transfected with miR-1-3p inhibitor and siRNA of E2F5 and PFTK-1 respectively for 72 h, inhibitor-NC and siControl served as respective negative control. **a** The effect of concomitant knockdown of miR-1-3p and E2F5 or PFTK1 on LnCap cells growth rates as measured by MTS assays. **b** and **c** Western blot analysis of E2F5 and PFTK1 protein levels in indicated cells. GAPDH was used as the loading control. **d** The effect of concomitant knockdown of miR-1-3p and E2F5 or PFTK1 on LnCap cells proliferative ability as determined by colony formation assays. **e-f** The proportion of indicated cells in distinct cell cycle phases as identified by FACS. Results were plotted as the mean ± SEM of three independent experiments, with at least three replicates in each independent experiment. (*P < 0.05, **P < 0.01)

To further explore whether miR-1-3p targeting of E2F5 and PFTK1 is responsible for inhibiting proliferation and cell cycle progression of PCa cells, we performed loss-of-function experiments by co-transfecting siE2F5/siPFTK1 or control along with the miR-1-3p inhibitor or inhibitor-NC into LNcaP cells. Relative quantification of the MTS assay showed that concomitant knockdown of miR-1-3p and E2F5 or PFTK1 reverses the inhibitory effects of either E2F5 or PFTK1 silencing alone (Fig. 5a). This phenomenon were further confirmed by WB and colony formation assays (Fig. 5b-d). Consistently, FACS was applied to analyze the distribution of cell cycle phases. LNcaP cells displayed a decreased proportion of the G1 phase and an elevated percentage of S phase after combined treatment with siE2F5/PFTK1 and miR-1-3p inhibitor, compared to miR-1-3p inhibitor transfected alone (Fig. 5e-f). These results collectively suggest that E2F5 and PFTK1 are a functional targets of miR-1-3p-induced suppression of PCa cell proliferation and cell cycle progression.

### MiR-1-3p suppresses the proliferation of PCa cells in the nude mice model

**Fig. 6:**
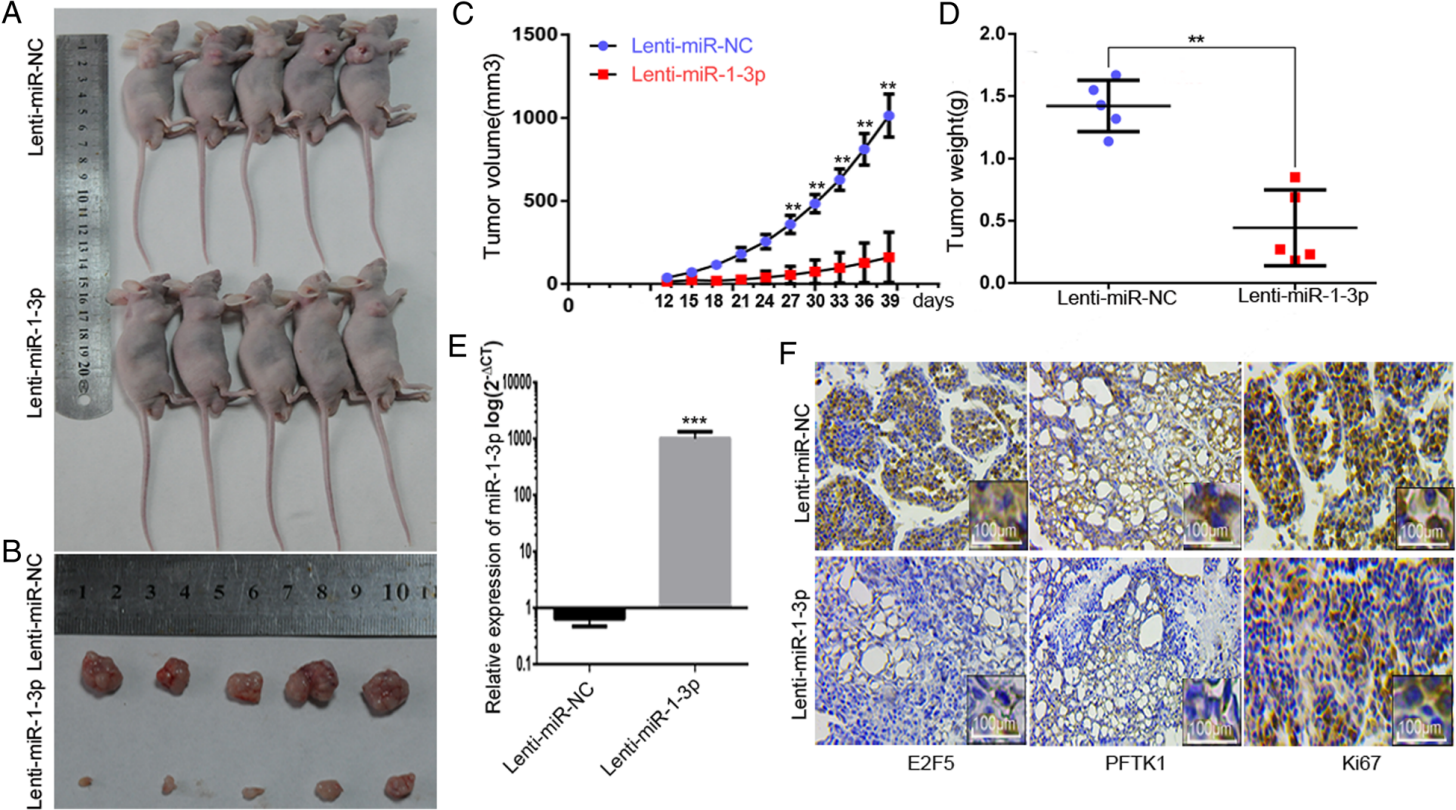
MiR-1-3p inhibits PCa tumor xenograft growth in vivo. **a** Representative photograph of tumor formation and (**b**) tumors excised 40 days after inoculation of stably transfected cells into nude mice. **c** Tumor volume was measured using a Vernier caliper on the indicated days. **d** Tumor weight in the miR-NC and miR-1-3p treated groups. **e** The relative expression of miR-1-3p in xenograft tumor tissue were identifed by RT-qPCR. **f** Immunohistochemical analysis of Ki-67,E2F5 and PFTK1 in xenografts tumors of miR-NC and miR-1-3p treated groups. Scale bar: 100 μm. (*P < 0.05, **P < 0.01)

To verify the therapeutic potential of miR-1-3p in vivo, LNcaP cells were injected into BALB/c nude mice in a xenograft model. To manipulate miR-1-3p levels, LNcaP cells were infected by the lentivirus expressing miR-1-3p before inoculation. Lenti-miR-NC served as a negative control. As expected, overexpression of miR-1-3p induced a dramatic reduction in tumor growth and volume in vivo compared with the miR-NC-treated group (Fig. 6a-c). Consistently, tumor weights were also significantly reduced in the Lenti-miR-1-3p-treated group (Fig. 6d). The relative expression of miR-1-3p in xenograft tumor tissue was verified by RT-qPCR (Fig. 6e). Furthermore, immunohistochemical staining of Ki-67 to assess tumor cell proliferation revealed a reverse correlation between the miR-1-3p levels and the expression of E2F5 and PFTK1 protein and cell proliferation (Fig. 6f). Taken together, these data indicate that the miR-1-3p may exert a significant inhibitory effect on tumorigenesis by repressing E2F5 and PFTK1 in vivo*.*

## Discussion

Accumulating studies have described that miRNA can function as tumor suppressors or oncogenes by regulating target gene expression levels in various human cancers [5, 26]. Multiple miRNAs have been observed to be involved in several crucial processes in PCa, such as cell proliferation, apoptosis, invasion and metastasis [9, 12, 14, 15, 17]. The tumor suppression activity of miR-1-3p during tumorigenesis has been well-characterized in several cancers, including colorectal cancer [20], bladder cancer [22] and prostate cancer [18, 19]. However, the underlying molecular mechanisms by which miR-1-3p modulates PCa carcinogenesis and the clinical significance of miR-1-3p in PCa patients remain poorly understood. In this study, we observed that downregulation of miR-1-3p is a frequent occurrence in PCa tissues and cell lines. Low-level expression of miR-1-3p was significantly associated with a more aggressive tumor phenotype and a short progression-free survival time for patients with PCa. In functional studies, proliferation and colony formation of PCa cells in vitro, and tumor growth in vivo*,* were dramatically suppressed upon reintroduction of miR-1-3p. These findings suggest that miR-1-3p plays a crucial role in the proliferation and/or cell cycle progression of PCa.

It is known that proliferation is one of the most important hallmarks of malignant tumors, and is the foremost fatal factor directly correlated with mortality in human cancers. Therefore, the identification of proliferative and/or cell cycle progressive factors as well as exploration of the underlying molecular mechanisms involved in miR-1-3p regulation of PCa progression in tumor growth are critical. We predicted its target genes using publicly available online algorithms, and identified that E2F5 and PFTK1, which have been demonstrated to have an important role in the cell proliferation, are potential functional targets of miR-1-3p. In our study, it was suggested that miR-1-3p binds to a complementary site, which is conserved among most vertebrates on the 3′-UTR of E2F5 and PFTK1, resulting in down-regulation of its target genes E2F5 and PFTK1 expression in PCa cells, as determined by luciferase assays and Western blot analyses. In addition, we also demonstrated that both E2F5 and PFTK1 were functionally involved in miR-1-3p-mediated suppression of proliferation and cell cycle progression in PCa cells. In addition, an inverse correlation between the levels of miR-1-3p and mRNA expression of E2F5 and PFTK1 was evaluated in our PCa cell lines and tissues. These observations provide the first line of evidence, to the authors’ knowledge, that miR-1-3p mechanistically acts through the regulation of both E2F5 and PFTK1 in PCa. It has been observed that E2F transcription factor 5 (E2F5) and/or PFTAIRE Protein Kinase 1 (PFTK1, also known as CDK14) are upregulated in various types of human cancers, including prostate cancer [27–29]. Furthermore, patients with high E2F5 and/or PFTK1 expression are associated with a more aggressive tumor phenotype [30, 31]. These results are consistent with our findings that miR-1-3p downregulation is associated with a more aggressive and/or poor prognostic PCa phenotype. These results are consistent with our findings that miR-1-3p downregulation is associated with a more aggressive and/or poor prognostic PCa phenotype.

Moreover, as mentioned above, E2F5 belongs to E2F family and is well-known for its role in cell proliferation and cell cycle progression by binding pocket proteins in the G1 phase [24]. Furthermore, previous studies have shown that E2F5 is negatively regulated by multiple miRNAs, such as miR-34a [32], miR-613 [33] andmiR-128-2 [34]. PFTK1 is a novel member of the Cdc2 family and can regulate the expression of cyclins and the cell cycle [25]. In addition, related studies have demonstrated that PFTK1 protein either activated or was involved in Wnt signaling and promoted migration and invasion [29]. It does appear, therefore, that in our PCa cells, miR-1-3p modulates cell proliferation via regulation of E2F5 and PFTK1. In our study, we observed further that silencing E2F5 and PFTK1 largely mimicked the proliferation and cell cycle progression-inhibiting effect of miR-1-3p overexpression. Concomitant knockdown of miR-1-3p and E2F5 and PFTK1 substantially reversed the inhibitory effects of silencing either E2F5 or PFTK1 alone. These results support our theory that E2F5 and PFTK1 are predominant mediators of miR-1-3p suppression of PCa cell proliferation and cell cycle progression, suggesting that loss of function of miR-1-3p may result in an enhanced expression of E2F5 and PFTK1 and, in turn, the susceptibility of cells to proliferation. Similar to our study, Zhang et al. reported that tci-miR-1-3p is involved in cyflumetofen resistance by targeting TCGSTM4 in Tetranychus cinnabarinus [35]. Frederico et al. also found that miR-1-3p participate in several alterations of TRIM63/FBXO32 gene/protein expression related to the pathophysiology of DM, including soleus muscle atrophy [36]. Moreover, Shang and colleagues demonstrated that miR-1-3p suppresses the invasion and migration of bladder cancer by up-regulating SFRP1 expression [22]. Clearly, our results, together with the findings of other groups, indicate that miR-1-3p may target multiple proteins that function spatiotemporally or in cooperation with different cellular processes.

Although we have shed new light on the molecular mechanism responsible for miR-1-3p in PCa progression, the detailed mechanism by which miR-1-3p is downregulated, such as through DNA promoter methylation [37], interaction with long noncoding RNA [38] or metabolic disorders (such as Hyperglycaemia, Hyperlipemia) induction [39], still need to be elucidated in future studies. More importantly, E2F activity is regulated by the retinoblastoma (Rb) “pocket” protein family members Rb, p107, and p130, which bind and inhibit E2F5 and recruit repressive factors to E2F-driven promoters [40]. However, mutation or genetic ablation of the Rb gene occurs commonly in prostate cancers and leads to dysfunction of RB-E2F pathway and increased proliferation [41]. On the other hand, the protein p107 contains another growth suppression domains that interactions with CDK/ cyclin complexes [42]. And interesting that CDK phosphorylation of p107 weakens the p107 C-terminal –E2F5–association [40]. Therefore, future studies are warranted to further elucidate the relationship among miR-1-3p, PFTK1(CDK14), E2F5 and Rb pocket protein, and discover crucial miR-1-3p -target negative regulation pairs with network topological importance and evaluate their clinical significance in human PCa [43].

## Conclusions

In this study, we investigated the potential role of miR-1-3p in PCa progression and its underlying mechanisms. The results suggest that downregulation of miR-1-3p plays an important role in PCa cell proliferation through the regulation of the cell cycle-related genes E2F5 and PFTK1. These results suggested that miR-1-3p functions as a novel cell cycle regulator and tumor suppressor in prostate cancer and could be employed as a new prognostic marker for and/or an effective therapeutic target against PCa.

## Additional files

Additional file 1: **Table S1.** miR-1-3p expression in multivariable analysis of both the TCGA database and Tongji cohort. (DOCX 18 kb)

Additional file 2: **Figure S1.** Knockdown or induction of miR-1-3p expression in LnCap cells was confirmed by RT-qPCR. GAPDH served as a loading control.The results were plotted as the mean ± SEM of three independent experiments, with at least three replicates in each independent experiment (*P < 0.05, **P < 0.01.). (TIF 94 kb)

Additional file 3: **Figure S2.** Knockdown the expression of E2F5 and PFTK-1 were analysed by Western blot. GAPDH served as a loading control. (TIF 226 kb)

## Abbreviations

3’-UTR: 3′-untranslated region
CDK2: Cyclin dependent kinase 2
CDK4: Cyclin dependent kinase 4
E2F5: E2F transcription factor 5
miR-1-3p: microRNA-1-3p
PCa: Prostate cancer
PFTK1: PFTAIRE Protein Kinase 1
