# Supplementary material for: The putative tumour suppressor miR-1-3p modulates prostate cancer cell aggressiveness by repressing E2F5 and PFTK1
Source: J Exp Clin Cancer Res. 2018 Sep 5;37:219. doi: 10.1186/s13046-018-0895-z (PMC6125869; doi:10.1186/s13046-018-0895-z)
Supplement: Supplementary file 1 — Table S1. miR-1-3p expression in multivariable analysis of both the TCGA database and Tongji cohort. (DOCX 18 kb) [file 13046_2018_895_MOESM1_ESM.docx]

| Analysis | Variable | Hazard Ratio (95%CI) | P-value |
| --- | --- | --- | --- |
| TCGA multivariable | age | 0.885 (0.479-1.637) | 0.698 |
|  | PSA | 4.113(1.434-11.800) | 0.009 |
|  | pT stage | 4.341(1.668-11.293) | 0.003 |
|  | lymphnode metastasis | 1.117(0.580-2.152) | 0.741 |
|  | miRNA-1-3p expression | 0.354(0.140-0.896) | 0.028 |
| Tongji Hospital group multivariable | age | 0.529(0.169-1.656) | 0.274 |
|  | PSA | 0.728(0.208-2.553) | 0.620 |
|  | pT stage | 1.917(0.463-7.945) | 0.369 |
|  | lymphnode metastasis | 1.594(0.461-5.509) | 0.461 |
|  | miRNA-1-3p expression | 0.209(0.059-0.739) | 0.015 |

**Table S1:** miR-1-3p expression in multivariable analysis of both the TCGA dataset and Tongji cohort

Hazard ratios (HRs) were estimated with a multivariate Cox proportional hazards model
