# Supplementary figures and images for: The putative tumour suppressor miR-1-3p modulates prostate cancer cell aggressiveness by repressing E2F5 and PFTK1
Source: J Exp Clin Cancer Res. 2018 Sep 5;37:219. doi: 10.1186/s13046-018-0895-z (PMC6125869; doi:10.1186/s13046-018-0895-z)

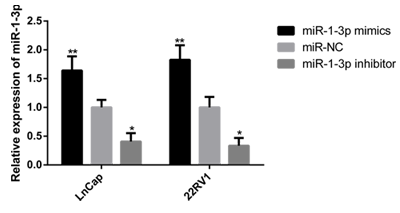

Supplement: Supplementary file 2 — Figure S1. Knockdown or induction of miR-1-3p expression in LnCap cells was confirmed by RT-qPCR. GAPDH served as a loading control.The results were plotted as the mean ± SEM of three independent experiments, with at least three replicates in each independent experiment (*P < 0.05, **P < 0.01.). (TIF 94 kb) [file 13046_2018_895_MOESM2_ESM.tif]

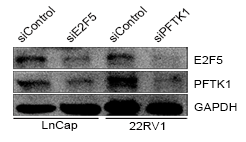

Supplement: Supplementary file 3 — Figure S2. Knockdown the expression of E2F5 and PFTK-1 were analysed by Western blot. GAPDH served as a loading control. (TIF 226 kb) [file 13046_2018_895_MOESM3_ESM.tif]
